# Post-mortem forensic application of proteomics on human ribs: Investigating the phenomenon of vital reaction

**DOI:** 10.1007/s00414-025-03519-w

**Published:** 2025-05-30

**Authors:** Nicola Galante, Daniele Capitanio, Manuela Moriggi, Laura Mangiavini, Riccardo D’Ambrosi, Alessio Battistini, Riccardo Zoja

**Affiliations:** 1 Section of Legal Medicine of Milan, Dipartimento di Scienze Biomediche per la Salute, University of Milan, Via Luigi Mangiagalli 37, 20133 Milan, Italy; 2 Department of Biomedical Sciences for Health, University of Milan, Milan, Italy; 3 IRCCS Ospedale Galeazzi – Sant’Ambrogio, Milan, Italy; 4 Department of Biomedical, Surgical and Dental Health Sciences, University of Milan, Milan, Italy

**Keywords:** Forensic proteomics, Vital reaction, Bone healing, Bone marrow, Carbonic anhydrase 2

## Abstract

The research upon vital reactions is a hot topic in forensic pathology. However, there are very few studies which focus on the bone tissue and scientific data are currently limited to conventional histopathology. The skin and the muscle are also faster than the bone in the healing process. Therefore, the possibility to establish the timing of skeletal traumatic injury is difficult especially for short survival times (*peri-mortem* interval). For these reasons, bone marrow may represent a dynamic and potentially useful substrate for the identification of bone lesion vitality. Furthermore, novel omics techniques such as the untargeted proteomics could really improve the investigation of reliable forensic markers. This study provides the application of proteomics on human bone marrow of traumatized ribs with the purpose to *a)* define a significant pattern of vital reaction on broken ribs *versus* undamaged ones; *b)* evaluate the proteomic changes over different known survival times; *c)* assess proteomic differences among resuscitation fractures *versus* other types of rib traumas (e.g., vehicle and train crashes, falling from heights). The trauma group significantly (*q* < 0,001) overexpresses acute-phase inflammatory proteins, different extracellular matrix proteins, and bone-related specific proteins. Noteworthy, some of these proteins show very interesting patterns such as complement C9 which provides the best significant results over time, fibrinogen which statistically increases starting from 4 h after trauma, and carbonic anhydrase 2 which is linearly overexpressed in the first 12 h. There are no statistical differences between resuscitation fractures *versus* other types of rib blunt injuries.

**Supplementary Information:**

The online version contains supplementary material available at 10.1007/s00414-025-03519-w.

## Introduction

According to Madea et al. the question whether an injury was sustained during life or not is one of the most important topics in forensic medicine. The term “vital reaction” describes effects in, at or by the body mostly following trauma and allowing the conclusion that the trauma occurred during life [1]. This is of utmost importance, especially in cases where multiple injuries occur within a short survival period, among conditions of physical abuse, or in the context of potential interference due to iatrogenic injuries.

Blunt force chest injuries with rib fractures are very frequently reported in forensic routine caseworks [2, 3]. Furthermore, one individual may show multiple blunt rib injuries which have been inflicted over a short period of survival time. Resuscitation procedures are often observed and complicate further the medicolegal examination [4, 5]. However, the definition of the lesion vitality on human bone is currently very limited [6, 7]. This issue is even more difficult among cases with early survival times [8].

Following the fracture, indirect healing begins, which scholastically consists of four steps with considerable overlap between these stages [2, 9, 10]. Mesenchymal stem cells coming from the surrounding tissues and bone marrow (BM) play a pivotal role in the bone healing process [10]: at first, hematoma formation starts soon after fracture and it is supported by mesenchymal cells, macrophages and inflammatory cells which form and clot the temporary frame for subsequent healing. Then, primary fibrocartilaginous (soft without mineralized cartilage) callus provides provisional stability within the first two weeks after trauma. A fibrin-rich-granulation tissue is first developed by fibroblasts and endothelial cells; then, mesenchymal cells differentiate and chondrogenesis begins to occur laying down a collagen-rich fibrocartilaginous.

Bony (hard) callus formation is regulated by fibroblast which secrete the matrix constituents such as collagen, elastic and mesh fibers, and glycoproteins. They start their differentiation into osteoblasts and the soft callus begins to undergo endochondral ossification within 30–40 days. Finally, bone remodeling continues for months to years; specifically, this process is continuously guided by the activity of osteoblasts and osteoclasts.

From a microscopical point of view [6], an acute hemorrhage with fibrin deposits and polymorphonuclear cells at the point of fracture can be generally seen within the first 2 days. After 3 days, there are fibroblasts, mesenchymal cells and gradual development of granulation tissue with necrosis of the bone adjacent to the fracture becomes more evident. The soft callus can be documented from 7 to 14 days, when the osteoid matrix begins to be deposited. Then, woven bone is remodeled by osteoblasts and osteoclasts into lamellar bone since the callus reaches its maximum size (3 weeks after trauma). Finally, the hard callus can be appreciated by the evidence of periosteal and endochondral ossification (> 4 weeks). Additionally, such healing stages are even more complex to investigate on dry bones [11].

In the light of the above, the application of proteomics on a novel and dynamic substrate such as the red bone marrow (rBM) represents a promising approach for forensic rib examination with special regard to the significance of its lesion vitality [12, 13]. Therefore, this study aims to *a)* identify the proteomic expression on red bone marrow of human ribs that were damaged with fracture (lesion vitality); *b)* evaluate the proteomic changes over different known survival times; *c)* assess proteomic differences among resuscitation fractures *versus* other types of rib traumas (e.g., vehicle and train crashes, falling from heights).

## Material and methods

### Study design

This study enrolled prospectively individuals that underwent full forensic autopsy. All the bodies were stored at 4 °C until the autopsy. Post-mortem interval (PMI) was always known for the trauma group (deaths witnessed/medically certified); for the control group, PMI was estimated from on-site investigation but always < 24 h. Specifically, the research included cases with displaced rib fractures due to known blunt force trauma (e.g., train collision, traffic accidents, falling from height and procedure of cardiopulmonary resuscitation) and with different survival times. Cardiopulmonary resuscitation (CPR) was performed manually, mechanically (LUCAS) or both sequentially; it did not exceed 45 min within which the emergency team obtained return of spontaneous circulation (ROSC). The selection of the ribs was morphologically based on the highest hemorrhagic infiltration which could be detected macroscopically. All the cases underwent a temporal stratification according to their survival times, as follows: t < 1 h (7); t = 1 h (4); t = 2 h (4); 4 < t < 10 h (5); 12 < t < 24 h (2); t > 24 h up to 96 h (3).

On the other hand, the absence of both skeletal and visceral injuries of the head, neck and trunk was chosen as the only inclusion criterion to enroll the controls; no control underwent resuscitation procedure as described in the emergency medical service reports. Sampling for controls was conventionally taken on the 4 th right rib on the anterior axillary line.

Cases and controls shared the same exclusion criteria such as signs of decomposition (i.e., greenish skin discoloration, post-mortem skin slippage, marbling, visceral flaccidity and opacity), children and adolescents (i.e., < 18 years old), and no or few anamnestic information related to the health conditions of the deceased.

The main features of the two cohorts are summarized in Tables 1 and 2 (Tab. 1; Tab. 2).

**Table 1:** Main features of cases that have been enrolled in this study

COHORT OF CASES						
N°	Age	Sex	Cause of death	Modality of death	Survival time	Area of sampling
1	53	M	Chronic ischemic heart disease	Natural (*me*CPR performed* for 24 min)	t < 1 h	5° right rib on anterior axillary line
2	44	F	Blunt force polytraumatic injuries	Train collision	t < 1 h	5° left rib on anterior axillary line
3	58	F	Blunt force polytraumatic injuries	Vehicle car accident	t < 1 h	5° right rib on anterior axillary line
4	25	M	Blunt force polytraumatic injuries	Vehicle car accident	t = 7 h	4° left rib on the emiclavear line
5	27	M	Blunt force polytraumatic injuries	Motorbike accident	t = 72 h	3° left rib on the paravertebral line
6	69	F	Blunt force polytraumatic injuries	Pedestrian being hit by car	t = 2 h	5° right rib on the anterior axillary line
7	52	F	Single inguinal stab wound	Acute bleeding (*ma* + *me*CPR performed* for 43 min)	t = 5 h	3° right rib on the emiclavear line
8	42	F	Blunt force polytraumatic injures	Falling from height	t = 2 h	2° left rib on the emiclavear line
9	86	M	Iatrogenic cardiac tamponade	Natural (*ma*CPR performed* for 40 min)	t = 1 h	4° left rib on the emiclavear line
10	48	M	Blunt force polytraumatic injuries	Motorbike accident	t = 1 h	5° right rib on the emiclavear line
11	89	F	Chronic ischemic heart disease	Natural (*ma*CPR performed* for 8 min)	t = 1 h	3° right rib on the emiclavear line
12	54	M	Blunt force polytraumatic injuries	Vehicle car accident	t < 1 h	2° left rib on the anterior axillary line
13	74	M	Blunt force polytraumatic injuries	Vehicle car accident	t = 10 h	9° right rib on the middle axillary line
14	60	F	Blunt force polytraumatic injuries	Bike accident	t = 2 h	4° right rib on the emiclavear line
15	30	M	Blunt force polytraumatic injuries	Pedestrian being hit by car	t = 24 h	5° right rib on the anterior axillary line
16	74	F	Blunt force polytraumatic injuries	Pedestrian being hit by car	t = 1 h	6° left rib on the paravertebral line
17	26	M	Blunt force polytraumatic injuries	Falling from height	t < 1 h	2° right rib on the emiclavear line
18	57	F	Blunt force polytraumatic injuries	Vehicle car accident	t = 4 h	9° left rib on the paravertebral line
19	46	M	Hanging	Asphyxia (*ma* + *me*CPR performed* for 35 min)	t = 6 h	6° right rib on the emiclavear line
20	46	M	Dilatative cardiomyopathy	Natural (*ma*CPR performed* for 19 min)	t = 2 h	3° left rib on the emiclavear line
21	81	M	Myocardial infarction	Natural (*ma*CPR performed* for 14 min)	t = 96 h	6° right rib on the anterior axillary line
22	37	M	Blunt force polytraumatic injuries	Bike accident	t < 1 h	4° left rib on the anterior axillary line
23	57	M	Blunt force polytraumatic injuries	Worker being crushed by forklift	t = 72 h	5° left rib on the paravertebral line
24	51	F	Blunt force polytraumatic injuries	Falling from height	t < 1 h	5° right rib on the middle axillary line
25	90	M	Blunt force polytraumatic injuries	Pedestrian being hit by car	t = 21 h	6° left rib on the posterior axillary line

^*^CPR: cardiopulmonary resuscitation *ma* = manually, *me* = mechanically (LUCAS). Mean age: 55,04 years; ratio M:F = 1,5:1

**Table 2:** Main features of controls that have been enrolled in this study

COHORT OF CONTROLS				
N°	Age	Sex	Cause of death	Modality of death
A	61	M	Opioid acute intoxication	Narcotism
B	72	F	Hanging	Asphyxia
C	41	M	Drug acute intoxication	Narcotism
D	26	F	Hanging	Asphyxia
E	66	M	Cut injuries on the wrists	Acute bleeding
F	48	M	Myocardial infarction	Natural
G	82	M	Myocardial infarction	Natural
H	54	M	Hanging	Asphyxia
I	63	F	Chronic ischemic heart disease	Natural
L	35	M	Hanging	Asphyxia
M	65	M	Chronic ischemic heart disease	Natural
N	62	M	Chronic ischemic heart disease	Natural
O	33	M	Hanging	Asphyxia
P	72	F	Drug acute intoxication	Narcotism
Q	47	M	Opioid acute intoxication	Narcotism

Mean Age: 55,13 years; ratio M:F = 2,75:1. All the samples have been conventionally collected from the 4 th right rib

### Tissue sampling

The traumatized ribs were cut by rib shears at a distance of 3 cm from the displaced fracture foci of the cases. Likewise, a 3 cm-long piece of undamaged ribs was collected from the controls. Soft tissues around each sample were mechanically removed, so that to obtain homogenous specimens of 5–7 g. All the samples were immediately frozen and stored at—80 °C until further preparation for the protein isolation.

### rBM preparation and proteomic analysis

The rib pieces were kept frozen throughout the bone marrow extraction process by keeping them in contact with a surface at—20 °C. The ribs were opened from the fracture traumatic site for cases and the medial iatrogenic fracture site for controls using forceps, the bone marrow was removed by scraping it with a scalpel and weighed. Bone marrow samples (50 mg) were suspended in 500 µL lysis buffer (2% SDS, 100 mM Tris–HCl pH 7.6, 0.1 M DTT, and 1% phenylmethanesulfonylfluoride) and sonicated on ice until completely dissolved. After incubation at 95 °C for 3 min, lysates were clarified by centrifugation at 16,000 × g for 10 min at 20 °C. Protein quantitation with 2-D Quant-kit protein assay (Cytiva, Little Chalfont, UK) was then performed. A detailed description of Label-Free Liquid Chromatography with Tandem Mass Spectrometry (LC–MS/MS) is reported in the Supplementary Material S1. Protein extracts (100 µg for each sample) were processed following the filter-aided sample preparation (FASP) protocol [14]. Two technical replicates for each sample were acquired. Mass spectra were analyzed using MaxQuant software (Max Planck Institute of Biochemistry, Munich, Germany, version 1.6.17.0) [15]. Spectra were searched by the Andromeda search engine against the *Homo sapiens* Uniprot UP000005640 sequence database (82,493 proteins, release March, 2024). Protein identification required at least one unique or razor peptide per protein group. Quantification in MaxQuant was performed using the built-in extracted ion chromatogram (XIC)-based label-free quantification (LFQ) algorithm using fast LFQ [16].

### Statistical analysis

Statistical analyses were performed using Perseus software (v. 2.0.11, Max Planck Institute of Biochemistry, Martinsried, Germany) [17]. For each experimental group, proteins identified in at least 70% of samples were considered. For statistical analysis, a Student’s t-test or an ANOVA test followed by a Tukey multicomparison analysis was applied, with a Benjamini–Hochberg FDR-corrected *p*-value threshold (*q*-value) of 0,05. Functional enrichment analysis of altered proteins was conducted using the g:Profiler public web server [18]. The g:GOSt algorithm was applied to functionally profile protein lists, identifying the overrepresentation of Gene Ontology Biological Process (GO:BP) terms and Reactome biological pathways.

## Results

**Fig. 1:**
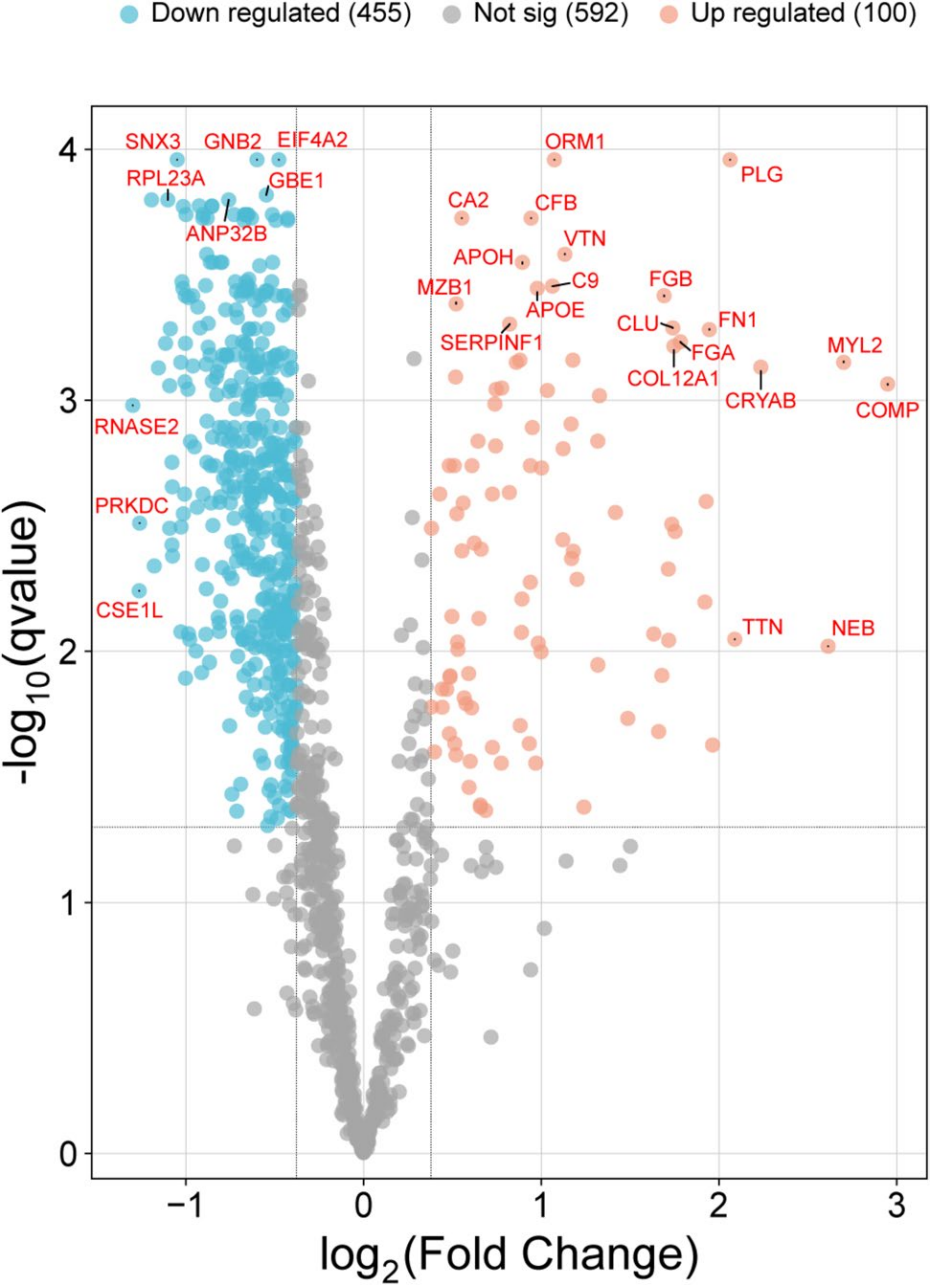
Volcano plot graphically describing the identified proteins (up- and downregulated) with their statistical significance in the slot all the cases (traumatic cohort) *versus* all the control (non-traumatic cohorts)

**Fig. 2:**
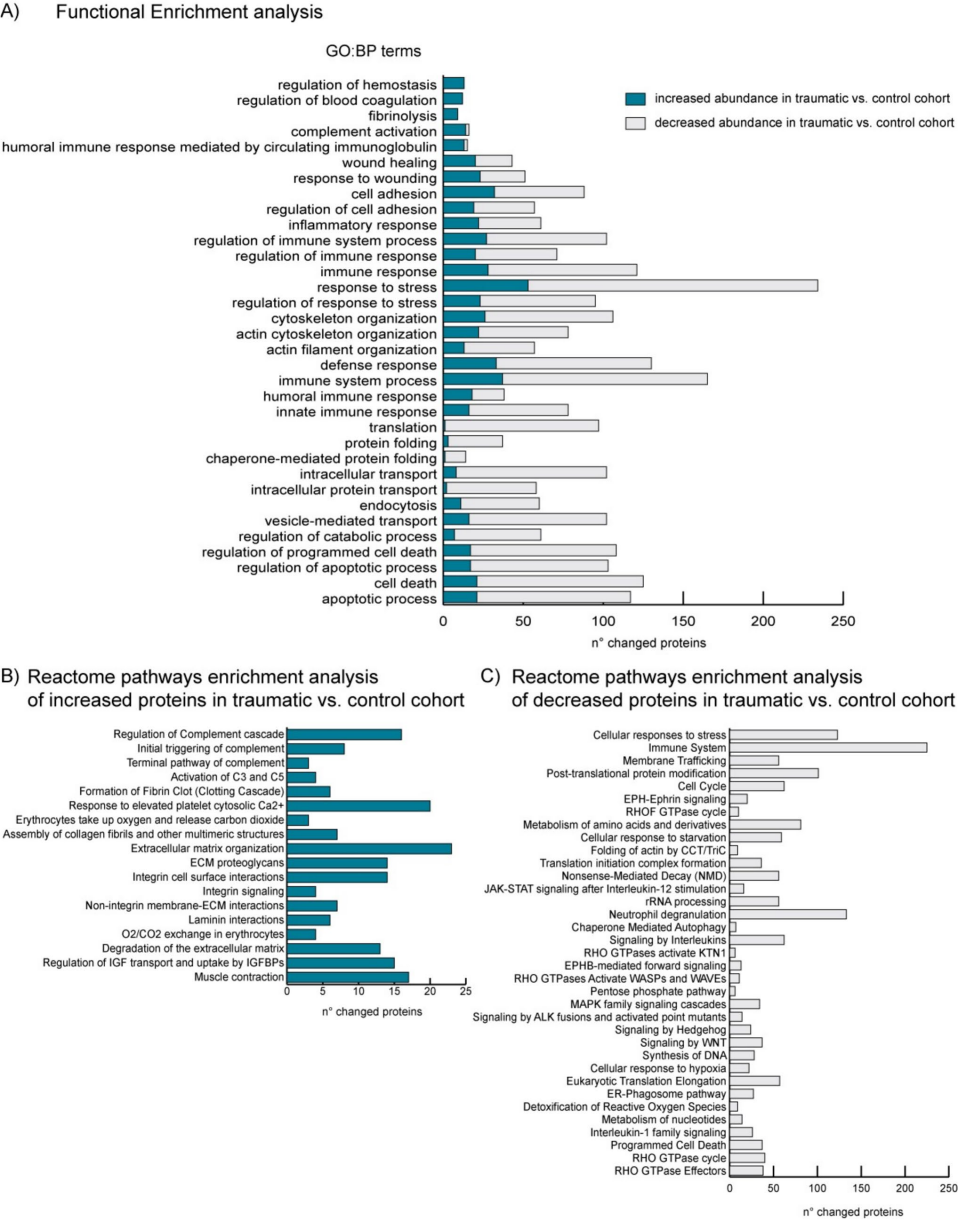
Functional enrichment analysis of differentially expressed proteins in the traumatic cohort compared to controls. **A** List of significantly overrepresented Gene Ontology biological process (GO:BP) terms (Fisher’s one-tailed test with Benjamini–Hochberg FDR, p < 0.05). The bar chart represents the number of proteins with increased (blue bars) or decreased (grey bars) abundance. **B** List of Reactome pathways overrepresented in the subset of proteins with increased abundance. The bar chart shows the number of increased proteins associated with each pathway. **C** List of Reactome pathways overrepresented in the subset of proteins with decreased abundance. The bar chart shows the number of decreased proteins associated with each pathway

Proteomic profiles were obtained from 40 individuals that included 25 cases with rib fractures (traumatic cohort) and 15 controls without any traumatic injuries of the chest (control cohort). A total of 1147 proteins were identified, and the abundance of 555 proteins significantly (Student’s t-test, *q* < 0,05) differed between the two groups, specifically 100 were upregulated and 455 downregulated (fold change cutoff = 1,3), as shown in the volcano plot (Fig. 1). Among these, 39 exhibited significant differences with fold changes > 2 between the groups. Functional enrichment analysis identified Gene Ontology Biological Process terms that were significantly overrepresented (Fisher’s one-tailed test with Benjamini–Hochberg FDR, p < 0.05) in the altered protein dataset, including hemostasis and coagulation, complement activation, cell adhesion, cytoskeletal organization, immune response, protein folding, endocytosis, intracellular transport, and apoptosis regulation (Fig. 2A). Enrichment analysis of upregulated proteins using the Reactome database revealed biological pathways associated with complement cascade activation, hemostasis, collagen fiber assembly, extracellular matrix organization, integrin signaling, and muscle contraction (Fig. 2B). In contrast, downregulated proteins were mainly involved in response to stress, immune system processes, protein synthesis and folding, autophagy, apoptosis, and various intracellular and cell–cell signaling pathways (Fig. 2C).

**Fig. 3:**
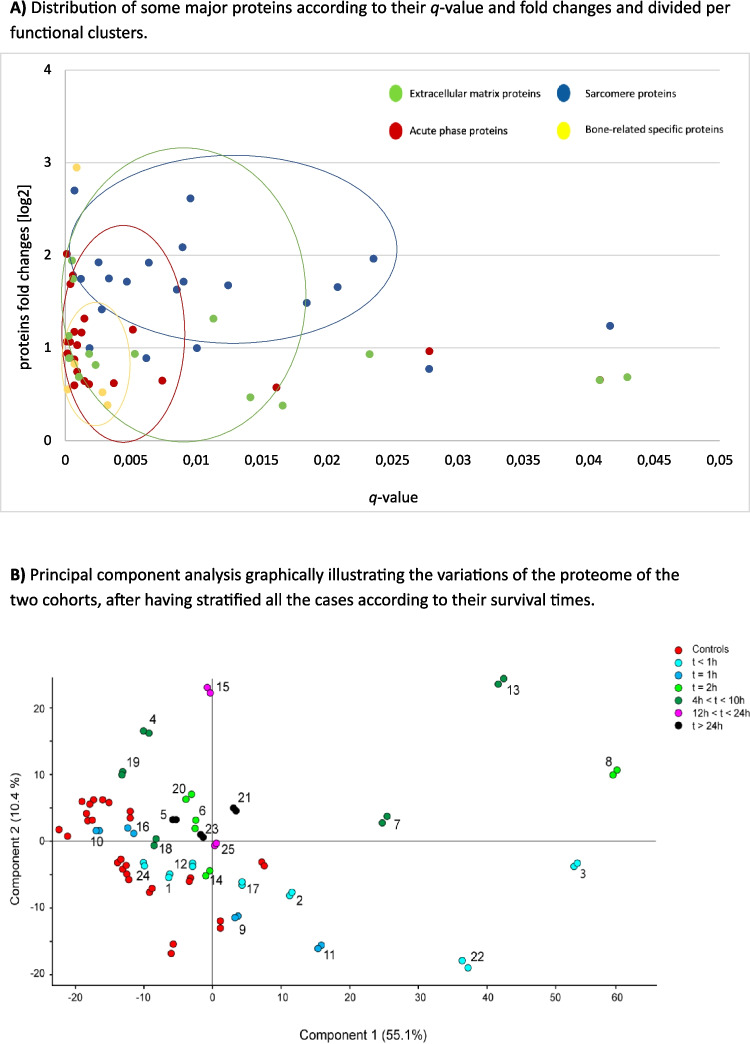
**A** Distribution of some major proteins according to their *q*-value and fold changes and divided per functional clusters. This diagram highlights that proteins belonging to the same cluster show similar behaviors; moreover, the clusters are intersected suggesting a functional cooperation among different proteins. **B** Principal component analysis graphically illustrating the variations of the proteome of the two cohorts, after having stratified all the cases according to their survival times. Note that each sample has two technical replicates which were almost all completely identical

Upregulated and statistically significant proteins were classified into 5 clusters according to their functional roles, as reported in Table 3 (Tab. 3). The abundance of these proteins depending on the significance is graphically detailed in a scatter plot with different colors each of which corresponds to the functional clusters (Fig. 3A).

**Table 3:** List of proteins divided per functional clusters with highest abundance and significance; *q*-values < 0,001 are highlighted in bold

	Protein	Gene	log_2_ fold change	*q*-value
ACUTE PHASE PROTEINS (APPs)	Plasminogen	PLG	2,016	**0,00011**
	Fibrinogen alfa chain	FGA	1,782	**0,00058**
	Fibrinogen beta chain	FGB	1,690	**0,00038**
	Fibrinogen gamma chain	FGC	1,317	0,00145
	Alpha-2 antiplasmin	SERPINF2	1,200	0,00516
	C4b-binding protein alpha chain	C4BPA	1,177	**0,00069**
	Histidine-rich glycoprotein	HRG	1,167	0,00124
	Alpha-1 acid glycoprotein 1	ORM1	1,072	**0,00011**
	Complement component C9	C9	1,062	**0,00035**
	Ig gamma-3 chain C region	IGHG3	1,033	**0,00091**
	Ig gamma-4 chain C region	IGHG4	0,968	0,02781
	Complement factor B	CFB	0,942	**0,00018**
	Beta-2 glycoprotein 1	APOH	0,892	**0,00028**
	Serum amyloid P-component	APCS	0,745	**0,00090**
	Kininogen-1	KNG1	0,648	0,00740
	Complement C1q subcomponent subunit C	C1QC	0,643	0,00145
	Protein AMBP	AMBP	0,622	0,00370
	Complement C4-A	C4 A	0,609	0,00182
	Complement factor H	CFH	0,597	**0,00068**
	Complement C5	C5	0,576	0,01613
SARCOMERE PROTEINS (SPs)	Myosin regulatory light chain 2	MYL2	2,700	**0,00070**
	Nebulin	NEB	2,613	0,00955
	Titin	TTN	2,088	0,00896
	Myosin-binding protein C, slow-type	MYBPC1	1,963	0,02355
	Myosin-7	MYH7	1,927	0,00253
	Troponin T, slow skeletal muscle	TNNT1	1,920	0,00637
	Myosin light chain 3	MYL3	1,752	0,00333
	Tropomyosin alpha-3 chain	TPM3	1,748	0,00119
	Mysosin-2	MYH2	1,716	0,00904
	Troponin I, slow skeletal muscle	TNNI1	1,714	0,00470
	Myosin-1	MYH1	1,678	0,01244
	Desmin	DES	1,659	0,02081
	Myosin light chain 1/3, skeletal muscle isoform	MYL1	1,632	0,00853
	Myosin regulatory light chain 2, skeletal muscle isoform	MYLPF	1,486	0,01845
	Tropomyosin beta chain	TPM2	1,416	0,00279
	Calsequestrin-1	CASQ1	1,239	0,04162
	Filamin-C	FLNC	1,000	0,00186
	Tropomyosin alpha-1 chain	TPM1	0,998	0,01006
	Myosin light chain 6B	MYL6B	0,890	0,00619
	Alpha-actinin-2	ACTN2	0,774	0,02781
EXTRACELLULAR MATRIX PROTEINS (ECMPs)	Fibronectin	FN1	1,945	**0,00052**
	Collagen alpha-1 (XII) chain	COL12 A1	1,745	**0,00060**
	Decorin	DCN	1,317	0,01131
	Vitronectin	VTN	1,131	**0,00026**
	Collagen alpha-2 (VI) chain	COL6 A2	0,938	0,00182
	Collagen alpha-1 (VI) chain	COL6 A1	0,937	0,00531
	Biglycan	BGN	0,932	0,02324
	Collagen alpha-3 (VI) chain	COL6 A3	0,879	**0,00069**
	Tenascin	TNC	0,819	0,00233
	Basement membrane-specific heparan sulfate proteoglycan core protein	HSPG2	0,738	0,00103
	Lumican	LUM	0,686	0,04295
	Collagen alpha-1 (XVIII) chain	COL18 A1	0,656	0,04084
	Laminin subunit beta-2	LAMB2	0,656	0,04162
	Collagen alpha-2 (IV) chain	COL4 A2	0,467	0,01413
	Laminin subunit gamma-1	LAMC1	0,381	0,01662
BONE-RELATED SPECIFIC PROTEINS (BSPs)	Cartilage oligomeric matrix protein	COMP	2,948	**0,00086**
	Alpha-2 HS-glycoprotein	AHSG	0,858	**0,00070**
	Carbonic anhydrase 2	CA2	0,552	**0,00018**
	Vitamin D-binding protein	GC	0,524	0,00283
	Band 3 anion transport protein	SLC4 A1	0,398	0,02509
MISCELLANEOUS	Alpha-crystallin B chain	CRYAB	2,234	**0,00073**
	Clusterin	CLU	1,739	**0,00051**
	Carbonic anhydrase 3	CA3	1,122	0,00155
	Apolipoprotein E	APOE	0,977	**0,00035**
	Protein 4.1	EPB41	0,776	**0,00089**
	Hemoglobin subunit delta	HBD	0,661	0,00392

**Fig. 4:**
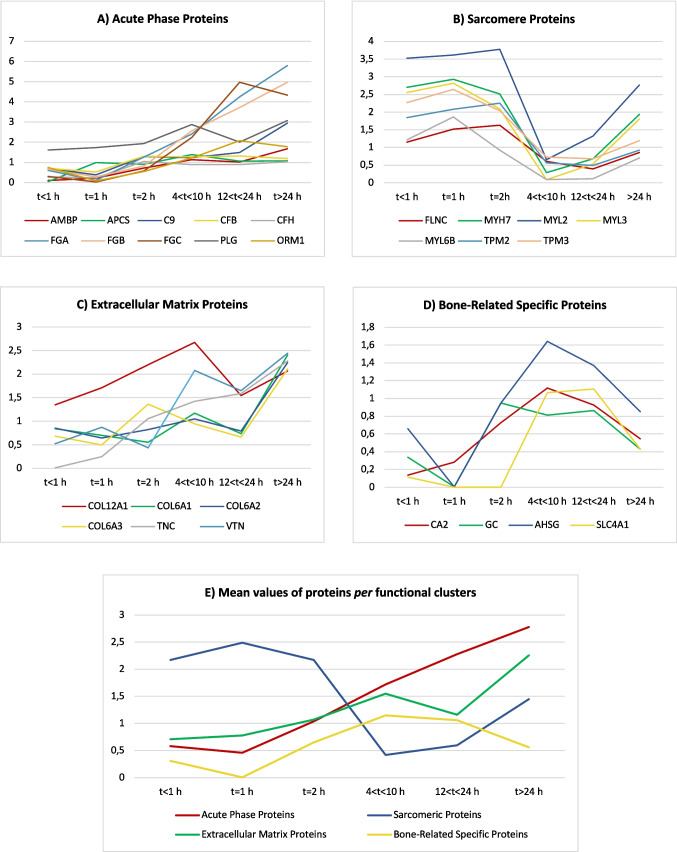
Variations of the proteome over the survival time and categorized by functional clusters. **A** acute phase proteins; **B** sarcomere proteins; **C** extracellular matrix proteins; **D** bone-related specific proteins; **E** mean values of proteins *per* functional cluster. Time slots are stratified according to the survival times, as follow t < 1 h; t = 1 h; t = 2 h; 4 < t < 10 h; 12 < t < 24 h; t > 24 h up to 96 h. The diagrams only show significant proteins (*q* < 0,05) in at least one time slot. Please, refer to Table 3 for all the proteins’ acronyms reported in this figure

After the stratification of the cases according to their survival times, 440 proteins were identified as statistically significant (ANOVA + Tukey test, *q* < 0,05) and differentiating from the control group. Principal component analysis of these proteins indicated that the traumatic cases had profiles distinguishable from those of controls, with the latter showing very small proteome changes which were also quite close to the subgroup with the shortest survival time (< 1 h) (Fig. 3B). The variations of the proteome over the survival times are graphically illustrated and categorized by functional clusters (Fig. 4A-E). Some proteins showed very interesting patterns such as complement C9 which provided the best significant results over time, fibrinogen (alfa, beta and gamma chains) which statistically increased starting from 4 h after trauma, and carbonic anhydrase 2 (CA2) which was linearly overexpressed in the first 12 h.

No statistical differences emerged between resuscitation fractures versus other types of rib injuries.

## Discussion

This study showed that traumatized rBM exhibits a specific and time-dependent proteomic response following rib fractures. A significant overexpression of acute-phase proteins, extracellular matrix components, and bone-specific proteins was observed in the trauma group compared to controls. These findings support the concept of rBM as a dynamic tissue capable of revealing vital reactions even within short survival times.

Many proteins belonging to the acute phase such as factors of the complement activation, coagulation and hemostasis were identified. These proteins also increased over the survival time along with the magnification of the inflammation, as demonstrated by the functional analysis (biological pathways). Hemostasis and coagulation proteins initiate the fracture healing process by forming a fibrin clot, which stabilizes the injury site and provides a matrix for cellular infiltration [6]. These proteins also release signaling molecules that recruit immune and progenitor cells. The complement system, activated early after injury, modulates inflammation, enhances phagocytosis of debris, and promotes osteogenic cell recruitment and differentiation [19]. Together, these systems start to orchestrate the transition from inflammation to tissue regeneration during bone repair [10].

Interestingly, rBM overexpressed, especially in the first 24 h after trauma, several complement factors of both the classic and alternative pathways at different levels and with high statistical significance, which suggests that this biological process can be referred as a general condition of vital reaction. Noteworthy, some of these proteins showed very interesting patterns such as complement C9 which provided the best significant results over time, according to its biological functions. Thus, C9 is a terminal component of the complement system, forming the membrane attack complex (MAC). In fracture repair, C9 contributes to the clearance of pathogens and damaged cells, indirectly supporting a controlled inflammatory environment necessary for bone regeneration [20]. Furthermore, fibrinogen statistically increased starting from 4 h after trauma**;** beyond its pathophysiological role in hemostasis, fibrinogen provides a scaffold for cell migration and releases bioactive peptides that modulate inflammation and promote osteoblast differentiation [21].

The proteomic analysis also demonstrated a pivotal role of proteins belonging to extracellular matrix organization, cell adhesion, cytoskeletal organization and collagen fiber assembly. rBM significantly overexpressed collagen VI starting from the first hours after injury, with a marked increment after 24–48 h. During fracture repair, it forms the organic matrix of the callus, guiding mineralization and providing mechanical strength. It also influences cell adhesion, migration, and differentiation of hematopoietic elements for the acute phase [22]. Collagen XII, decorin, tenascin, vitronectin, and COMP were also upregulated but pose more specific pathophysiological considerations. Noteworthy, collagens and other ECM proteins participate in the establishment of the primary soft callus which gradually substitutes the hematoma [6, 9, 10]. This finding corresponds to the results of the current research and highlights a *fluid* cooperation of different proteins (APPs and ECMPs) in the bone healing process. Therefore, these ECM proteins can be considered as suitable rBM markers of early lesion reaction in the forensic setting since they show significant increases over time with only selected expressions among other tissues.

rBM overexpressed after traumatization high-specific proteins related to bone metabolism which include carbonic anhydrase 2, alpha-2 HS-glycoprotein, vitamin D-binding protein, and COMP. CA2 also showed an interesting parabolic trend with an increment over the first 12 h and likewise a decrement up to 96 h after trauma, as shown in Fig. 4D. This enzyme is one of sixteen forms of human carbonic anhydrases [23]. Carbonic anhydrase catalyzes reversible hydration of carbon dioxide and interacts with band 3 anion transport protein. Defects in CA isoform 2 are associated with osteopetrosis, cerebral calcifications and renal tubular acidosis [24]. Specifically, CA2 deficiency is the paradigm osteopetrosis featuring failure of osteoclasts to resorb bone due to inability to acidify their pericellular space [25]. CA2 loss-of-function leads clinically to bone fragility and pathological fractures. Furthermore, bone healing in individuals suffering from osteopetrosis is impaired [26], thus suggesting a central role of CA2 in this biological process.

On the other hand, AHSG is a fetuin protein which still shows obscure roles. However, it can inhibit calcification as well as osteogenesis in bone [27]. Furthermore, osteoclasts are stimulated by GC [28]. According to these considerations, rBM immediately starts to upregulate some highly specific proteins related to bone metabolism to promote the osteoclastic resorption of injured hydroxyapatite with a maximum peak at 12 h after trauma. This finding is of utmost importance since it postulates that CA2, SLC4 A1, and AHSG may be useful to distinguish early vital reactions in bone tissue due to the activation of osteoclasts in the context of bone turnover.

Before concluding, bioinformatic analysis revealed two important proteins which are used in routine forensic pathology for vitality definition such as glycophorin A (GLYA) [29–31] and aquaporin-3 (ACQ3) [1, 32, 33]. However, they did not show a statistical significance between the trauma group and the controls. Similarly, many proteins involved in the mechanisms of adhesion (ICAM3, MCAM, NCAM1, PECAM1, and VCAM1) [34] were not identified as significant, thus imposing great caution in their use on bones as markers of vital reaction.

## Limitations of the study

Limitations include the selection of well-preserved dead bodies with known survival times, specific rBM analyses, and a sample size which can be further extended. Despite differences in cardiopulmonary resuscitation methods, the proteomic profiles did not significantly diverge, suggesting that the biological response of red bone marrow is predominantly influenced by the vitality status and survival time rather than the nature of the trauma itself. However, the potential influence of CPR methods, intensity, and clinical context cannot be entirely excluded and deserves further investigation.

## Conclusive remarks

Consistent with the initial hypotheses of the project, injured rib bone marrow exhibited significant proteomic changes, indicating activation through statistically significant modulation of several expected proteins (i.e., regulation of complement cascade, extracellular matrix organization and degradation) and specific bone ones. Overall, a marked up-regulation of proteins associated with the inflammatory process and bone tissue repair was observed.

- The proteomic profile was dynamic in the first 96 h post-trauma, with a rise in acute phase proteins and extracellular matrix proteins, while bone-specific molecules peaked uniquely between 12 and 24 h.
- A panel of significant proteins (e.g., C9, fibrinogen, collagen VI, CA2) may be extremely useful in forensic caseworks to both identify a vital reaction on rib fractures (*ante-mortem* trauma) and estimate the time interval between the traumatic mechanism and death. These potential biomarkers can minimize the limbo of the *peri-mortem* period and enable forensic pathologists to solidly assess a recent rib fracture or more different fractures in the same dead body.
- The traumatic mechanism itself did not affect the proteome, suggesting that vital reaction and survival time are key factors.

## Supplementary Information

Below is the link to the electronic supplementary material.

ESM 1 (DOCX 115 KB)

## Data Availability

The datasets generated and/or analyzed during the current research are available from the corresponding author on reasonable request.
