# Supplementary material for: Post-mortem forensic application of proteomics on human ribs: Investigating the phenomenon of vital reaction
Source: Int J Legal Med. 2025 May 30;139(5):2593–604. doi: 10.1007/s00414-025-03519-w (PMC12354616; doi:10.1007/s00414-025-03519-w)
Supplement: Supplementary file 1 — (DOCX 115 KB) [file 414_2025_3519_MOESM1_ESM.docx]

Label-Free Liquid Chromatography with Tandem Mass Spectrometry (LC-MS/MS).

Protein extracts (100 µg for each sample) were processed following the filter-aided sample preparation (FASP) protocol [14]. Each sample was deposited in a Microcon-30 kDa centrifugal filter unit (Merck Millipore, Burlington, MA, USA) and washed by centrifugation at 14,000 g for 15 min with 200 µL of UA buffer (8 M urea, 0.1 M Tris/HCl, pH 8.5). Samples were carbamydomethylated in 100 µL of 50 mM iodoacetamide in UA buffer for 20 min, then washed three times in 100 µL UA buffer followed by three washes in 100 µL of 50 mM ammonium bicarbonate in water. Filters were incubated with sequence grade trypsin (Promega, Madison, WI, USA) for 16 h at 37 °C using a protein:trypsin ratio of 50:1. After acidification with trifluoracetic acid and desalting on C18 tips (Zip-Tip C18 micro, Merck Millipore, Burlington, MA, USA), peptide samples were vacuum concentrated, reconstituted in HPLC mobile phase A (0.1% formic acid) and separated on a Dionex UltiMate 3000 nano-LC System equipped with an EASY-Spray column ES902, 25 cm x 75 µm ID packed with Thermo Scientific Acclaim PepMap RSLC C18, 3 µm, 100 Å (Thermo Fisher Scientific) using an increasing gradient of mobile phase B (0.1% formic acid in acetonitrile 20/80, v/v) at a flow rate of 0.300 μL/min. Peptide mixtures were electrosprayed into an Orbitrap Exploris 240 (Thermo Fisher Scientific) mass spectrometer. Two technical replicates for each sample were acquired. One blank was run between samples to prevent sample carryover. MS spectra were collected over an m/z range of 375 – 1500 Da at 120,000 resolutions, operating in the data dependent mode, cycle time 3 sec between masters scans. HCD was performed with collision energy set at 35 eV. Polarity: positive. Mass spectra were analyzed using MaxQuant software (Max Planck Institute of Biochemistry, Munich, Germany, version 1.6.17.0) [15]. The maximum allowed mass deviation was set to 6 ppm for monoisotopic precursor ions and 0.5 Da for MS/MS peaks. Enzyme specificity was set to trypsin/P, and a maximum of two missed cleavages was allowed. Carbamidomethylation was set as a fixed modification, while N-terminal acetylation and methionine oxidation were set as variable modifications. Spectra were searched by the Andromeda search engine against the *Homo sapiens* Uniprot UP000005640 sequence database (82,493 proteins, release March, 2024). Protein identification required at least one unique or razor peptide per protein group. Quantification in MaxQuant was performed using the built-in extracted ion chromatogram (XIC)-based label-free quantification (LFQ) algorithm using fast LFQ [16]. The required FDR was set to 1% at the peptide, 1% at the protein, and 1% at the site-modification levels, and the minimum required peptide length was set to 7 amino acids.
